# Does Cholangiovenous Reflux Cause Cholangitis?

**DOI:** 10.1155/1990/86530

**Published:** 1990

**Authors:** Henry A. Pitt

**Affiliations:** Blalock 688 Johns Hopkins Hospital 600 N. Wolfe Street Baltimore MD 21205 USA


### REFERENCES

1. Broughan T.A., Sivak M.V. and Hermann R.E. (1985) The management of retained and recurrent common bile duct stones. *Surgery*, **98**, 748–751.
2. Classen M. and Demling L. (1974) Endosckopischl sphincterotomic der papilli Vateri und stein-extraktion aus dem ductus choledochus. *Dtsch. Med. Wschr.*, **99**, 496–7.
3. Cotton P.B. (1980) Non-operative removal of bile duct stones by duodenoscopic sphincterotomy. *Br. J. Surg.*, **67**, 1–5.
4. Worthley C.S., Watts J. McK. and Toouli J. (1988) Common duct exploration or endoscopic sphincterotomy for choledocholithiasis. *ANZ. J. Surg.*, **59**, 209–216.
5. Neoptolemos J.P., Carr-Locke D.L. and Fossard D.P. (1987) Prospective randomised study of pre-operative endoscopic sphincterotomy versus surgery done for common bile duct stones. *Bri. Med. J.*, **194**, 470–474.
6. Allen B., Shapiro H. and Way L.W. (1981) Management of recurrent and residual common duct stones. *Am. J. Surg.*, **142**, 41–47.
7. Escourrou J., Cordova J.A., Lazorthes F., Frexinos J. and Ribert A. (1984) Early and late complications after endoscopic sphincterotomy for biliary lithiasis with and without gallbladder *"in situ"*. *Gut*, 598–602.
8. Worthley C.S. and Toouli J. (1988) Gallbladder non filling: an indication for cholecystectomy after endoscopic sphincterotomy. *Br. J. Surg.*, **75**, 796–798.
9. Neoptolemos J.P., Carr-Locke D.L., Fraser I. and Fossard D.P. (1984) The management of common bile duct calculi by endoscopic sphincterotomy in patients with gallbladder *in situ*. *Br. J. Surg.* **71**, 69–71.
10. Martin D.F. and Tweedle D.E.F. (1987) Endoscopic management of common duct stones without cholecystectomy. *Br. J. Surg.*, **74**, 209–211.
11. Rosseland A.R. and Solhaug J.H. (1988) Primary endoscopic papillotomy (EPT) in patients with stones in the common bile duct and the gallbladder in situ: a 5–8 year follow-up study. *World. J. Surg.*, **12**, 111–116.
12. Classen M. and Hagenmuller F. (1984) Endoscopic biliary drainage. *Scand. J. Gastroenterology* **19**, (suppl 102) 76–83.
13. Sauerbruch T., Delius M., Paumgartner G., *et al* (1986) Fragmentation of gallstones by extracorporeal shockwaves. *N. Eng. J. Med.* **314**, 818–22.
14. Speer A.G., Webb D.R., Collier N.A., McHutchinson J., St. John D.J.B. and Clunie G.J. (1988) Extracorporeal Shockwave Lithotripsy and the management of common bile duct calculi. *Med. J. Aust.*, **148**, 590–5.

### DOES CHOLANGIOVENOUS REFLUX CAUSE CHOLANGITIS?

### ABSTRACT

*Stewart L, Pellegrini CA, Way LW. Cholangiovenous Reflux Pathways as Defined by Corrosion Casting and Scanning Electron Microscopy. The American Journal of Surgery 1988; 155: 23–8.*

**Using corrosion casting and scanning electron microscopy of the rat biliary tree, we investigated the site and size of the pathways that allow bacteria to reflux from bile to blood. Nonobstructed rat biliary trees were injected retrograde with methylmethacrylate resin at a constant rate of 0.04 ml/min to volumes of 40, 60, 80, 120, 160, and**

**200 μl. The infusion pressure was monitored and a pressure-volume curve was constructed. After polymerization and corrosion in 30 percent potassium hydroxide, the casts were examined with scanning electron microscopy. In addition, to identify the size of the reflux pathways, ceramic particles of 150 A, 1.7 μ, or 10 μ were added to the resin, and the studies were repeated. Finally, intact livers with casted biliary trees were processed and studied by scanning electron microscopy without corrosion.**

**Scanning electron microscopy demonstrated fine anatomic detail of the cholangiovenous reflux pathway. At 40 μl (20 cm water pressure) normal biliary radicals were filled. Between 40 and 80 μl (20 to 50 cm water pressure), the cast material refluxed from the bile ductules into the spaces of Mall and Disse and then into the hepatic sinusoids. Filling of sinusoids continued at volumes between 80 to 160 μl, and filling of collecting veins was seen above 160 μl. Particles of 1.7 μ and smaller readily refluxed, but there was no sinusoidal reflux of casting material that contained particles of 10 μ. Casting without corrosion showed that the liver parenchyma remained intact. There was no evidence of reflux across hepatocytes.**

**This study shows that cholangiovenous reflux occurs directly from bile ductules through the spaces of Mall and Disse into the hepatic sinusoids. The pathways measure between 1.7 and 10 μ. Since this is the path of least resistance, it may be of greater importance in the reflux of bacteria and toxins than other high-resistance pathways, for example, biliary canaliculi, tight junctions, or hepatocytes.**

### PAPER DISCUSSION

KEYWORDS: Corrosion cast, cholangiovenous reflux, cholangitis

Over 40 years ago, Mixter and his associates[1] demonstrated that cholangiovenous reflux occurs when contrast media is injected into the bile duct. Twenty years ago Huang *et al*[2] demonstrated in dogs that cholangiovenous reflux of *Escherichia coli* is directly related to biliary pressure. These investigators also showed that cholangiolymphatic reflux of bacteria occurs at the same levels of intrabiliary pressure which cause cholangiovenous reflux. In 1984 Yamamoto and Phillips[3] performed corrosion-cast experiments in rats and noted filling of periportal lymphatic spaces after biliary injection. One theoretical problem with this study, however, was the relatively high viscosity of the casting compound. Therefore, Stewart *et al*[4] have recently repeated these studies with a lower viscosity mixture which approximates the viscosity of bile.

The findings of Stewart *et al*[4] confirm the earlier observations of Yamamoto and Phillips[3]. These studies have demonstrated that cholangiovenous reflux in the rat progresses from the proximal bile ductules into 1) the spaces of Mall and Disse, 2) the hepatic sinusoids, and 3) the collecting veins. This process occurs without filling of bile canaliculi and without disruption of hepatocytes. Moreover, particles of 1.7 μ or smaller were able to reflux in this manner whereas particles of 10 μ size did not reflux. This observation is consistent with previous studies which have demonstrated that bacteria can reflux into both the hepatic veins and lymphatics while erythrocytes, which are 6 or 7 $\mu$ in diameter, are too large to reflux even at high pressures.

An interesting additional observation made by Stewart *et al*[4] was that the "resistance" to reflux varied as this process occurred progressively into 1) the spaces of Mall and Disse, 2) the hepatic sinusoids, and 3) the collecting veins. Resistance, as measured from pressure/volume relationships, increased as reflux progressed from the bile ductules into the spaces of Mall and Disse at pressures from 20 to 50 cm of water. Resistance then decreased to values observed during biliary ductal filling as reflux continued into the hepatic sinusoids at pressures from 50 to 80 cm of water. Resistance then fell to practically zero as reflux progressed into the hepatic collecting veins at pressures of approximately 80 cm of water.

These "resistance" data were obtained by retrograde injection into the common bile duct at a constant rate of 0.04 ml/min and correlated with electron microscopy. An interesting extension of these studies would be to measure resistance and observe reflux pathways at different flow rates. Clinically, cholangitis, and therefore reflux, is most likely to occur with rapid increases in intrabiliary pressure. The question remains, therefore, whether the reflex pathways observed by Stewart *et al*[4] would be altered by different flow rates. If the infusions had been more rapid, would the pathways have differed and would "high-resistance pathways" such as biliary canaliculi or hepatocytes have been involved?

Another interesting question is the relative contribution of cholangiovenous and cholangiolymphatic reflux to clinical cholangitis. The studies by Yamamoto and Phillips[3] and by Steward *et al*[4] both suggest that cholangiolymphatic reflux occurs before and at lower pressures than cholangiovenous reflux. This observation suggests that if intrabiliary pressures are raised to only moderate levels (20 to 50 cm of water) cholangiolymphatic reflux may be the only route for bacteria to gain access, via the thoracic duct, to the venous system. This scenario would be possible because at pressures below 50 cm of water Stewart *et al*[4] did not observe reflux into the hepatic sinusoids or collecting veins.

Another issue that must be considered is the relevance of these rat studies to man. Humans and many other species have a gallbladder, but the rat does not. One of the important functions of the gallbladder is to absorb water and, thereby, concentrate bile. In the face of distal biliary obstruction, the absorptive function of the gallbladder may actually moderate intrabiliary pressures and keep them below the hepatic secretory pressure. Thus, a species such as the rat, which does not have a gallbladder, may have reflux pathways that are different from those present in man. Confirmation of the studies by Steward *et al*[4] in a species with a gallbladder would add credence to their observations.

A third point that must be considered when interpreting these corrosion casting studies is the similarity of the casting compound to bile, Stewart *et al*[4] have attempted to improve upon the study by Yamamoto and Phillips[3] by lowering the viscosity of the casting compound. Stewart and her colleagues demonstrated quite nicely that the addition of inert ceramic particles of different sizes dramatically affected "reflux pathways." What would they have observed, however, had they also studied the effect of varying the osmolality of all of the casting compound?

For clinical cholangitis to occur two factors must be present: 1) increased intrabiliary pressure and 2) bacteria. The presence of bacteria in bile, however, may actually change its chemical character. Bacteria may secrete glycoproteins and enzymes. Bacterial enzymes may deconjugate both bilirubin and bile salts.

Deconjugated bile salts are more likely to diffuse into and damage cells and, thereby, increase their permeability. Another interesting study, therefore, would be to add deconjugated bile salts to the casting compound.

Finally, various bacteria may either enhance or impede cholangiovenous or cholangiolymphatic reflux. Possible enhancing mechanisms have been mentioned above. Could the bacterial production or mucus glycoproteins actually block reflux pathways? Alternatively, could the presence of *pili* on certain bacteria promote their attachment to ductal epithelial cells and, thereby, inhibit reflux? Thus, as with many good studies, the work of Stewart *et al*[4] may have raised more questions than were answered. Hopefully, future studies on the pathogenesis of cholangitis will address 1)the rate of pressure rise, 2)the influence of species difference, 3)the impact of bile composition, and 4)variable effects of different bacteria.

Henry A. Pitt
Blalock 688
Johns Hopkins Hospital
600 N. Wolfe Street
BALTIMORE, MD 21205
USA

### REFERENCES

1. Mixter, H.W., Rigler, L.G., and Gonzales-Oddone, M.V.(1947) Experimental studies on biliary regurgitation during cholangiography. *Gastroenterology*, **9**, 64–80
2. Huang, T., Bass, J.A. and Williams, R.D. (1969) The significance of biliary pressure in cholangitis. *Archives of Surgery*, **98**, 629–32
3. Yamamoto, K. and Phillips, M.J. (1984) A hitherto unrecognized bile ductular plexis in normal rat liver *Hepatology*, **4**, 381–85
4. Stewart, L., Pellegrini, C.A. and Way, L.W. (1988) Cholangiovenous reflux pathways as defined by corrosion casting and scanning electron microscopy. *American Journal of Surgery*, **155**, 23–27

